# Short-term deceleration capacity: a novel non-invasive indicator of parasympathetic activity in patients undergoing pulmonary vein isolation

**DOI:** 10.1007/s10840-024-01899-4

**Published:** 2024-08-20

**Authors:** Łukasz Zarębski, Piotr Futyma

**Affiliations:** 1 St. Joseph’s Heart Rhythm Center, Anny Jagiellonki 17, 35-623, Rzeszów, Poland; 2 University of Rzeszów, Rzeszów, Poland

**Keywords:** Atrial fibrillation, Pulmonary vein isolation, Short-term deceleration capacity, Short-term heart rate variability, Parasympathetic activity

## Abstract

**Background:**

Subtypes of atrial fibrillation (AF) can differ, and exact mechanisms in which patients benefit from the pulmonary vein isolation (PVI) remain not fully understood. During PVI, vagal innervation of the heart may also be affected. Thus, non-invasive methods of intraprocedural assessment of such PVI impact are sought.

**Methods:**

From 1-minute ECG recordings performed before and after PVI, we investigated short-term deceleration capacity (ST-DC) and short-term heart rate variability (ST-HRV) to determine their potential as indicators of parasympathetic activity before and after ablation.

**Results:**

In 24 consecutive patients with paroxysmal AF included in the study, there were a significant differences in ST-DC and ST-HRV parameters measured before and after PVI. After 3 months, patients with baseline ST-DC ≥ 7.5 ms were less likely to experience AF recurrence when compared to patients with baseline ST-DC < 7.5 ms (0% vs 31%, *p* = 0.0496). There were no differences in AF recurrence after 12 months of follow-up (36% vs 38%, *p* = 0.52).

**Conclusion:**

PVI leads to significant changes in ST-DC and ST-HRV, and these parameters can serve as indicators of vagal denervation after AF ablation. Patients with more prominent baseline ST-DC are less likely to experience AF recurrence during the post-PVI 3-month blanking period.

**Graphical Abstract:**

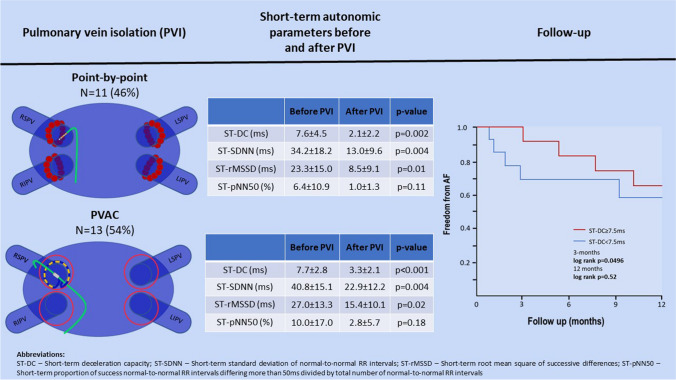

## Introduction

Atrial fibrillation (AF) is currently the most prevalent cardiac arrhythmia, and pulmonary vein isolation (PVI) has emerged as a well-established therapy [1, 2]. Nevertheless, the variability of AF subtypes leads to divergent PVI outcomes across heterogeneous cohorts of patients suffering from AF [3]. Two predominant autonomic influences, vagal and adrenergic, play major roles in shaping individual profiles of AF [4]. Vagal AF is characterized by male predominance, younger age, and minimal tendency to progress to permanent AF [3]. In these patients, increased parasympathetic tone leads to prominent bradycardia being trigger for a subsequent AF. This particular AF subtype often occurs during periods of rest or sleep, and may be triggered by other activities that enhance vagal tone, such as swallowing [3]. In contrast, adrenergic AF is usually associated with structural heart disease and heightened sympathetic activity. Impact of stress, physical exertion, or emotional triggers frequently provoke adrenergic AF episodes [4].

Increased autonomic activity during vagal AF episodes suggests that ablating the ganglionated plexi (GPs) area could diminish the burden of AF episodes through the attenuation of parasympathetic tone [5]. Several clinical studies have investigated the suppression of AF through GP ablation either independently or in combination with PVI [6–9]. The main conclusion from these studies was that GP ablation may slightly increase the efficacy of PVI; however, not to such extent as it was hoped. However, majority of these studies did not determine exactly the magnitude of parasympathetic influence. Therefore, phenotyping of the specific AF patient profiles in the setting of PVI and exact impact of cardiac autonomic modulation in AF subgroups of patients before and after catheter ablation would be of value. Different methods have been investigated to find the appropriate indicators for vagal tone activity. Initial studies based on respiratory-related variation of cardiac cycle showed that heart rate variability (HRV) may be used to assess autonomic nervous system activity [10]. However, it requires 24 h of Holter monitoring and dedicated software to be calculated. For this reason, its utility is limited for intraprocedural assessments which are urgently needed. This kind of evaluations may be performed with extracardiac vagal stimulation (ECVS) [11]. ECVS is performed to compare vagal response, such as sinus arrest and atrioventricular block before the procedure and after the procedure; when the lack of ECVS effects on cardiac rhythm should be observed after successful vagal denervation [12]. However, this method is also not perfect due to a requirement of catheter advancement to the vagal nerve area and general anesthesia [13]. Ideally, assessment of parasympathetic activity should be noninvasive and quickly available in the setting of the electrophysiology laboratory.Combining the advantages of these two noninvasive methods, we examined short-term deceleration capacity (ST-DC) and short-term heart rate variability (ST-HRV)—two easily available parameters, which do not require long-term monitoring. Recently, numerous studies have investigated the validity and reproducibility of ST-HRV recordings confirming its utility [14–19]. However, no role for ST-DC utilization has been up-to-date investigated. Previously, deceleration capacity (DC) was observed to be able to overcome some limitations associated with HRV such as elimination of artifacts, providing more stationary and reliable evaluations in long-term monitoring [20]. Thus, the goal of our study was to investigate an impact of PVI on these markers of dynamic autonomic activity with subsequent assessments of their correlation with AF recurrences.

## Methods

### Study design and data collection

Between April 2019 and March 2021, consecutive patients with paroxysmal AF undergoing PVI at our institution were included into the study. Demographic information, clinical history, and procedural details were systematically obtained from electronic medical records. Detailed clinical history, presence of pre-existing conditions, prior cardiac interventions, and medication histories, were analyzed. The study adhered to the ethical guidelines outlined in the Declaration of Helsinki. Informed consent for PVI was obtained from all patients.

### Calculation of ST-DC

Calculation of ST-DC was derived from the 1-minute monitoring of R-R intervals during sinus rhythm, recorded using the EP system (EP Tracer, Cardiotek, Maastricht, The Netherlands). Heartbeat intervals longer than the preceding interval were defined as anchors. R-R interval prolongations > 5% were excluded to avoid errors. Then, the signals X within the aligned segments were averaged. ST-DC was quantified using the equation ST-DC = 1/4 (*X*0 + *X*1 − *X* − 1 − X − 2). *X*0 and *X*1 are the averages of the anchor points and the following R-R intervals, while *X* − 1 and *X* − 2 are the averages of the 2 R-R intervals preceding the anchor points. These calculations were performed before and right after the PVI procedure.

### Calculation of ST-HRV

Calculation of ST-HRV was derived from the result of a 1-min monitoring of R-R intervals during sinus rhythm, recorded using the EP system (EP Tracer, Cardiotek, Maastricht, The Netherlands). The most common variables of ST-HRV were used.

#### Time-domain variables

- Standard deviation of normal-to-normal RR intervals (SDNN)—calculated by computing the standard deviation of all normal-to-normal RR intervals in the sample
- Root mean square of successive differences (rMSSD)—calculated by computing the square root of the mean of the squared differences between successive normal-to-normal RR intervals
- Proportion of success normal-to-normal RR intervals differing more than 50 ms divided by total number of normal-to-normal RR intervals (pNN50)—determined by counting pairs of successive normal-to-normal RR intervals differing by more than 50 ms and dividing this number by the total number of normal-to-normal RR intervals in the sample, expressed as a percentage.

#### Frequency-domain variables

- High frequency (HF) power: calculated by integrating the power spectral density in the range of 0.15 to 0.4 Hz.
- Low frequency (LF) power: calculated by integrating the power spectral density in the range of 0.04 to 0.15 Hz.
- Very low frequency (VLF) power: calculated by integrating the power spectral density in the range of 0.0033 to 0.04 Hz.
- LF/HF ratio: calculated by dividing the LF power by the HF power.

Power spectral density analysis was performed using a fast Fourier transform to decompose the RR interval series into its frequency components and to quantify the power distribution across the specified frequency bands.

All ST-HRV parameters were calculated before and right after PVI.

### Pulmonary vein isolation

Oral anticoagulation was taken continuously by patients before the procedure. Under local anesthesia, vascular access to the right femoral vein was obtained under ultrasound guidance [21]. Diagnostic four- or decapolar catheter was introduced in the coronary sinus. Under fluoroscopic guidance, a single transseptal puncture was performed in all cases. Heparin was administered until activated clotting time > 350 s was achieved. Using Carto (Biosense Webster, Diamond Bar, CA) or EP Navigator (Philips Medical Systems, Best, The Netherlands) systems a three-dimensional map of the left atrium (LA) was created. Next, a RF energy was delivered, starting from the right and moving to the left PVs ostia with either an open irrigated-tip catheter (Thermocool, Biosense Webster, USA; Flexability, Abbott, USA; CoolFlex, St. Jude Medical, USA) from the EP-Shuttle generator (Stockert GmbH, Freiburg, Germany); or with multi-electrode PVAC gold catheter (Medtronic, Minneapolis, MN, USA) using phased RF delivered from the multichannel generator (Genius, Medtronic, Minneapolis, MN, USA). For point-by-point (PBP) strategy, RF lesions were delivered at power settings between 30 and 70 W depending on the equipment and technique used. In general, the aim was to deliver high-power short duration applications (up to 8 s) at the posterior aspects of PVs. Conversely, moderate (30 W) to high (50 W) applications at slightly extended duration (up to 15 to 20 s each) were aimed near anterior aspects of PV ostia, aimed for unipolar signal modification and impedance drop > 10%. For multielectrode approach, 3 to 4 phased RF applications were performed for each PV with typical settings recommended by manufacturer (maximal power—10 W, maximal RF time—60 s). The endpoint was the dissociation of all PV potentials and loss of pace capture from the ablation line. Manual compression at the venous access site was applied in all patients, and postprocedural echocardiogram was performed to exclude pericardial effusion or other acute complications.

### Follow-up

Patients were monitored for at least 24 h after PVI and then discharged. Follow-up was scheduled which consisted of multiple ambulatory visits 4 weeks, 3 months, 6 months, and 12 months after the procedure. Patients were asked about their symptoms and had an anthropometric measurements and 12-lead ECG taken at each follow-up visit. A detailed history of any palpitations, episodes of AF, and hospitalizations for cardiac arrhythmias were collected. Recurrence of arrhythmia was defined as an episode of AF recorded on ECG lasting more than 30 seconds.

### Data analysis

Continuous variables were presented as mean ± standard deviation or median (interquartile range) based on the distribution. Categorical variables were expressed as percentages. Comparative analyses were conducted using *t*-tests for continuous variables and chi-square or Fisher’s exact tests for categorical variables. The Pearson correlation coefficient was used to calculate correlations between variables. A *p* value < 0.05 was considered to indicate statistical significance.

## Results

**Fig. 1:**
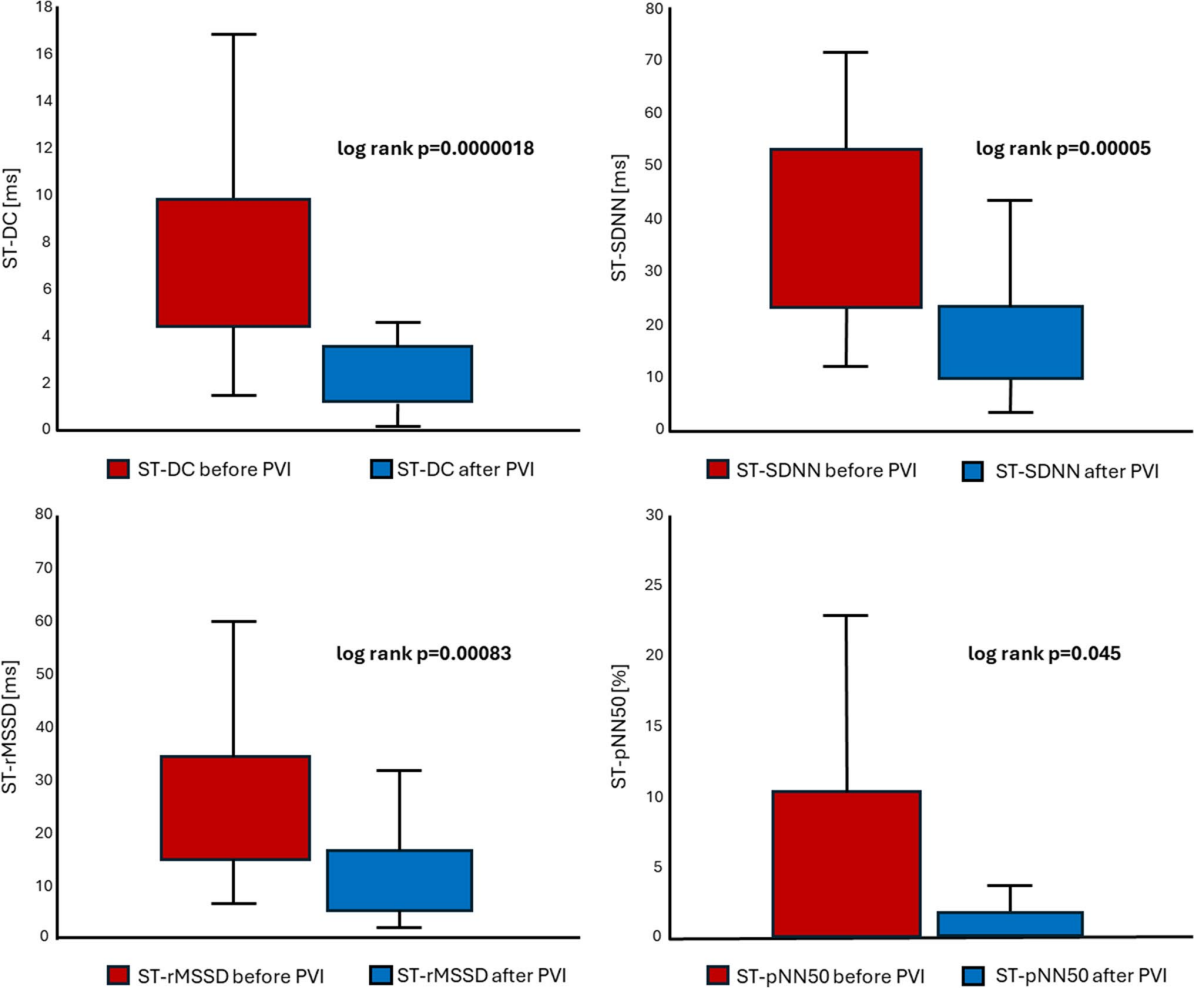
Alternations of ST-DC and time domain components of ST-HRV as a result of PVI. PVI, pulmonary vein isolation; ST-DC, short-term deceleration capacity; ST-pNN50, short-term proportion of success normal-to-normal RR intervals differing more than 50 ms divided by total number of normal-to-normal RR intervals; ST-rMSSD, short-term root mean square of successive differences; ST-SDNN, short-term standard deviation of normal-to-normal RR intervals

**Fig. 2:**
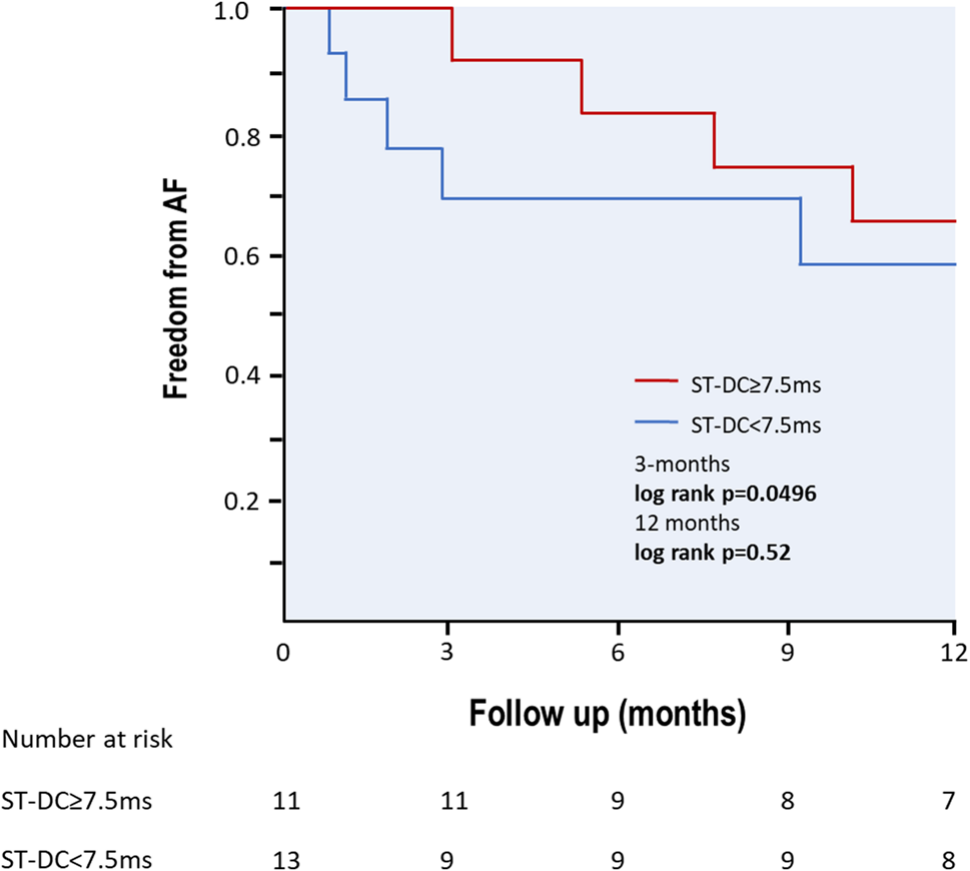
Kaplan–Meier curves comparing survival from AF in patients with ST-DC ≥ 7.5 ms and ST-DC < 7.5 ms measured before PVI. AF, atrial fibrillation; ST-DC, short-term deceleration capacity

The study included 24 consecutive patients (8 females, age 54 ± 11 years) with paroxysmal atrial fibrillation who underwent PVI. Eleven patients underwent PBP PVI (46%), and the remaining 13 patients (54%) underwent PVAC PVI. Clinical and demographical characteristics of the study population regarding PVI technique is presented in Table 1. The procedure time was shorter when PVI was performed using the PVAC approach compared to the PBP technique (86 ± 18 min vs 157 ± 54 min, *p* = 0.0002). The RF time was also shorter (980 ± 151 s vs 1401 ± 461 s, *p* = 0.007), as was the fluoroscopy time (1146 ± 585 s vs 1877 ± 699 s, *p* = 0.01). Moreover, heart rate (HR) increase after PVI was significantly greater in PBP group when compared to patients treated with PVAC (14 ± 12 bpm vs 3 ± 12 bpm, *p* = 0.03). There were no major complications. One minor event occurred in the PVAC group related to a malfunction of the PVAC catheter’s steering mechanism. In this case, the PVAC catheter was retracted uneventfully from the LA, and PVI was successfully completed with PBP approach.Comparison of ST-DC before and after PVI revealed a significant difference (pre-PVI vs post-PVI, 7.6 ms vs 2.8 ms; *p* = 0.0000018). Additionally, ST-HRV parameters in the time domain demonstrated notable alterations: SDNN (pre-PVI vs post-PVI, 37.8 ms vs 18.4 ms; *p* = 0.00005), rMSSD (pre-PVI vs post-PVI, 25.3 ms vs 12.2 ms; *p* = 0.00083), and pNN50 (pre-PVI vs post-PVI, 8.4% vs 2%; *p* = 0.045) (Fig. 1). Also, in the frequency domain, significant changes were observed in the LF (pre-PVI vs post-PVI, 667.5 ms^2^ vs 129.4 ms^2^, *p* = 0.002), VLF (pre-PVI vs post-PVI, 613.3 ms^2^ vs 154.1 ms^2^, *p* = 0.005), and LF/HF ratio (pre-PVI vs post-PVI, 5.4 vs 2.0, *p* = 0.02) components. For the HF component, a trend in alteration was observed, but without statistical significance (pre-PVI vs post-PVI, 166.6 ms^2^ vs 81.4 ms^2^, *p* = 0.12). Additionally, significant increase in HR was observed after PVI (pre-PVI vs post-PVI, 65.5 bpm vs 73.7 bpm; *p* = 0.016). In the PVAC group, there was a very weak correlation between the time of applications and ∆ST-DC (*r* = 0.16, *p* = 0.65). However, there was a strong negative correlation between the time of applications and changes in ST-HRV parameters: ∆SDNN (*r* =  − 0.85, *p* < 0.001), ∆rMSSD (*r* =  − 0.86, *p* < 0.001), ∆pNN50 (*r* =  − 0.79, *p* = 0.004), ∆HF (*r* =  − 0.75, *p* = 0.008), ∆LF (*r* =  − 0.9, *p* < 0.001), ∆VLF (*r* =  − 0.7, *p* = 0.02), ∆HF/LF (− 0.82, *p* = 0.002). In the PBP group, there was a weak correlation between the time of applications and ∆ST-DC (*r* = 0.3, *p* = 0.32). Unlike the PVAC group, we did not observe any significant correlations between the time of applications and changes in ST-HRV parameters: SDNN (*r* =  − 0.31, *p* = 0.3), ∆rMSSD (*r* = 0.19, *p* = 0.53), ∆pNN50 (*r* =  − 0.08, *p* = 0.79), ∆HF (*r* =  − 0.002, *p* = 0.99), ∆LF (*r* =  − 0.41, *p* = 0.16), ∆VLF (*r* =  − 0.04, *p* = 0.9), and ∆HF/LF (− 0.35, *p* = 0.24). Upon a 3-month follow-up, patients with a baseline ST-DC ≥ 7.5 ms were less likely to experience AF recurrence compared to those with baseline ST-DC < 7.5 ms (0% vs 31%, *p* = 0.0496). There was no difference in AF recurrences rates after 12 months (36% vs 38%, *p* = 0.52) (Fig. 2). No significant correlations between ST-HRV and AF recurrences during the follow-up period were observed. Clinical and demographical characteristics of the study population regarding AF recurrences are presented in Table 2.

**Table 1:** Clinical and demographical characteristics of the study population regarding PVI technique

	PVAC group (*n* = 13)	PBP group (*n* = 11)	*p* value
Males	8	8	0.56
Age (years)	54 ± 11	55 ± 12	0.93
BMI	28.6 ± 4.3	29.1 ± 3.6	0.77
Height (cm)	172 ± 9	171 ± 8	0.87
Weight (kg)	84 ± 10	86 ± 13	0.75
CHA2DS2-VASc	1.7 ± 1.5	1.3 ± 1.4	0.51
LVEF (%)	60 ± 9	59 ± 2	0.85
LA (mm)	41 ± 5	42 ± 5	0.78
Propafenone use	4 (31%)	3 (27%)	0.85
Amiodarone use	1 (8%)	2 (18%)	0.44
Sotalol use	3 (23%)	1 (9%)	0.36
Β-blocker use	4 (31%)	6 (54%)	0.24
Baseline HR (bpm)	64 ± 6	67 ± 11	0.25
∆HR after PVI (bpm)	3 ± 12	14 ± 12	0.03
Baseline ST-DC (ms)	7.7 ± 2.8	7.6 ± 4.5	0.49
∆ST-DC after PVI (ms)	− 4.3 ± 3.1	− 5.5 ± 5.4	0.24
Baseline SDNN (ms)	40.8 ± 15.1	34.2 ± 18.1	0.98
∆SDNN after PVI (ms)	− 17.9 ± 21.1	− 21.1 ± 22.3	0.37
Baseline rMSSD (ms)	27.0 ± 13.3	23.3 ± 15.0	0.99
∆rMSSD after PVI (ms)	− 11.6 ± 18.8	− 14.8 ± 19.1	0.25
Baseline pNN50 (%)	10.0 ± 17.0	6.4 ± 10.9	0.90
∆pNN50 after PVI (pp)	− 7.2 ± 18.9	− 5.8 ± 11.4	0.82

*BMI*, body mass index; *HR*, heart rate; *LVEF*, left ventricle ejection fraction; *LA*, left atrium; *pp*, percentage points; *PV*, pulmonary vein isolation; *ST-D*, short-term deceleration capacity; *SDNN*, standard deviation of NN intervals; *rMSSD*, root mean square of successive differences; *pNN50*, proportion of successive NN intervals differing more than 50 ms divided by the total number of NN intervals

**Table 2:** Clinical and demographical characteristics of the study population regarding AF recurrence

	AF recurrence (*n* = 9)	No AF recurrence (*n* = 15)	*p* value
Males	6 (67%)	10 (67%)	1.00
Age (years)	53 ± 11	55 ± 12	0.57
BMI	28.2 ± 3.9	29.1 ± 4.0	0.60
Height (cm)	172 ± 8	172 ± 9	0.97
Weight (kg)	83 ± 10	86 ± 12	0.61
CHA2DS2-VASc	1.2 ± 1.3	1.7 ± 1.5	0.50
LVEF (%)	57 ± 8	63 ± 3	0.15
LA (mm)	42 ± 5	41 ± 4	0.78
Propafenone use	3 (33%)	4 (27%)	0.73
Amiodarone use	2 (22%)	1 (7%)	0.26
Sotalol use	1 (11%)	3 (20%)	0.57
Β-blocker use	5 (56%)	5 (33%)	0.29
Baseline HR (bpm)	68 ± 7	64 ± 9	0.31
∆HR after PVI (bpm)	9 ± 14	11 ± 13	0.86
Baseline ST-DC (ms)	7.3 ± 3.8	7.8 ± 3.6	0.74
∆ST-DC after PVI (ms)	− 4.7 ± 5.6	− 5.7 ± 3.4	0.87
Baseline SDNN (ms)	32.1 ± 13.4	41.2 ± 17.8	0.22
∆SDNN after PVI (ms)	− 10.7 ± 18.6	− 15.5 ± 21.8	0.14
Baseline rMSSD (ms)	19.3 ± 8.8	28.9 ± 15.6	0.12
∆rMSSD after PVI (ms)	− 9.9 ± 13.2	− 13.0 ± 21.5	0.55
Baseline pNN50 (%)	9.4 ± 6.2	13.9 ± 11.7	0.19
∆pNN50 after PVI (pp)	− 1.7 ± 4.3	− 2.0 ± 20.3	0.26

*BMI*, body mass index; *HR*, heart rate; *LVEF*, left ventricle ejection fraction; *LA*, left atrium; *pp*, percentage points; *PVI*, pulmonary vein isolation; *ST-DC*, short-term deceleration capacity; *SDNN*, standard deviation of NN intervals; *rMSSD*, root mean square of successive differences; *pNN50*, proportion of successive NN intervals differing more than 50 ms divided by the total number of NN intervals

**Fig. 3:**
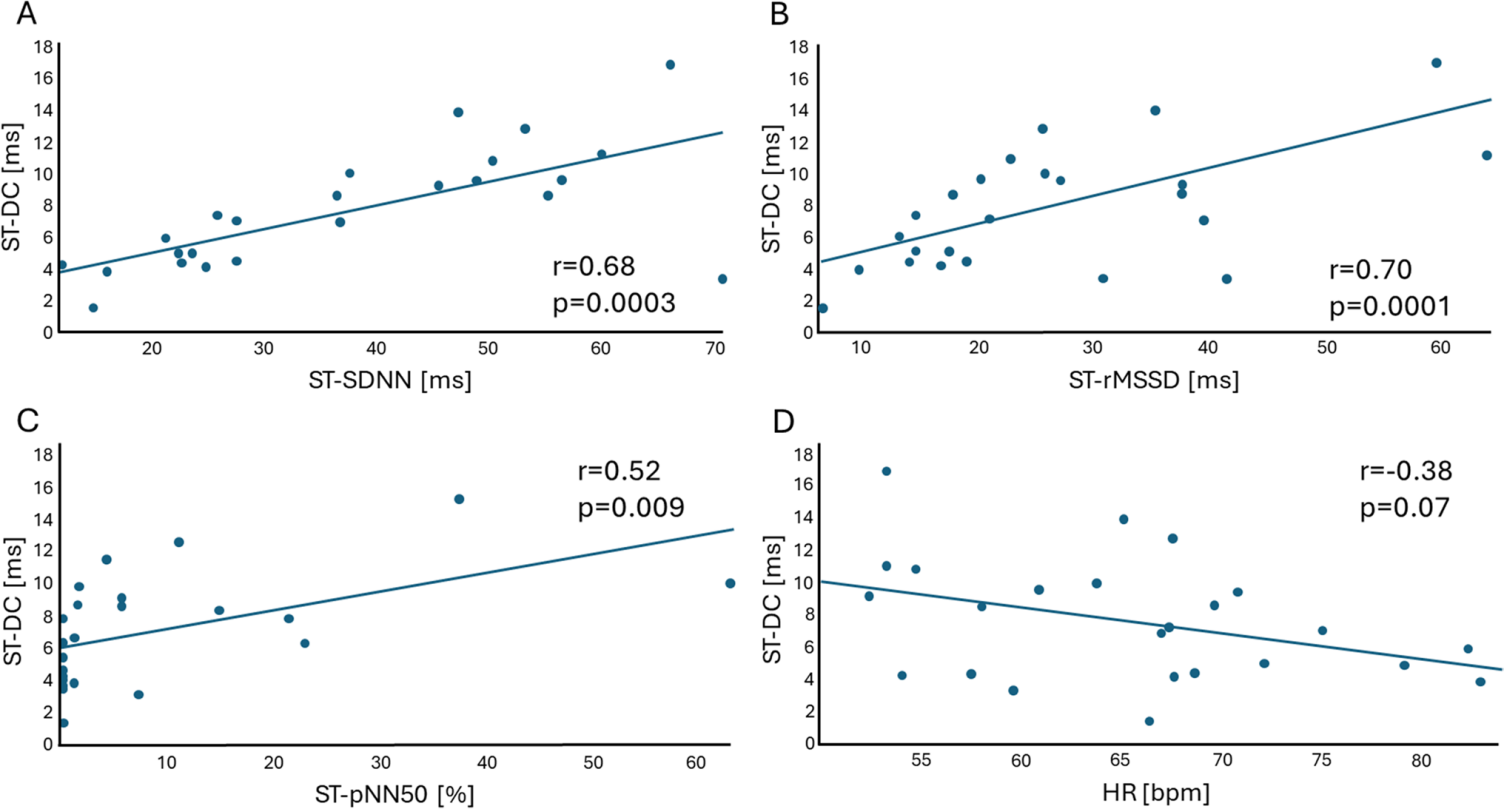
Scatterplots demonstrating the relationship between ST-DC, individual ST-HRV parameters, and HR measured before PVI. Panel **A** Demonstration of strong positive correlation between baseline ST-DC and ST-SDNN. Panel **B** Demonstration of strong positive correlation between baseline ST-DC and ST-rMSSD. Panel **C** Demonstration of moderate positive correlation between baseline ST-DC and ST-pNN50. Panel **D** Demonstration of weak negative correlation between baseline ST-DC and HR. ST-DC, short-term deceleration capacity; SDNN, standard deviation of normal-to-normal RR intervals; rMSSD, root mean square of successive differences; pNN50, proportion of success normal-to-normal RR intervals differing more than 50 ms divided by total number of normal-to-normal RR intervals

In the examination of the relationship between markers of parasympathetic activity, significant associations emerged between ST-DC, ST-HRV parameters, and heart rate (HR). Before PVI, strong positive correlations were identified between ST-DC and SDNN (*r* = 0.68, *p* = 0.0003), as well as between ST-DC and rMSSD (*r* = 0.70, *p* = 0.0001). Additionally, ST-DC demonstrated a moderate positive correlation with pNN50 (*r* = 0.52, *p* = 0.009). Conversely, a moderate negative correlation was observed between ST-DC and heart rate (HR) (*r* =  − 0.38, *p* = 0.07) (Fig. 3). Moreover, during data analysis, our study explored correlations between the shifts (∆) in ST-DC, ST-HRV, and HR. The results revealed the following associations: a positive correlation was observed between the ∆ST-DC and the ∆SDNN (*r* = 0.53, *p* = 0.008). In terms of frequency domain HRV parameters, significant associations were also observed. Before PVI, ST-DC showed a moderate positive correlation with HF (*r* = 0.46, *p* = 0.02) and VLF (*r* = 0.55, *p* = 0.005), and a weak positive correlation with LF/HF ratio (*r* = 0.33, *p* = 0.11). The LF component showed a strong positive correlation with ST-DC (*r* = 0.6, *p* = 0.002). Similarly, a strong positive correlation was identified between ∆ST-DC and the ∆rMSSD (*r* = 0.65, *p* = 0.0006), and a moderate positive correlation between ∆ST-DC and the ∆pNN50 (*r* = 0.45, *p* = 0.03). Contrarily, ∆ST-DC exhibited a moderate negative correlation with the ∆HR (*r* =  −0.59, *p* = 0.002), demonstrating the association between the decrease of ST-DC value and an increase in HR.

## Discussion

Parasympathetic activity has been recognized as one of the crucial factors in the pathogenesis of AF [22]. The balance between sympathetic and parasympathetic tone significantly influences the initiation and perpetuation of AF episodes. Such sympathetic-parasympathetic balance can be influenced by the catheter ablation when aiming for durable PVI [23]. These changes are usually reflected by HR acceleration frequently observed after successful PVI [23], and such shift in HR is exclusively visible during thermal-based catheter techniques for PVI [24–27]. Given the ongoing advent of non-thermal methods of AF ablation, which are likely to have less impact on cardiac parasympathetic activity [28], the autonomic innervation of the heart is an area of increasing interest, especially in the field of AF ablation outcomes in some specific subpopulations. However, despite to-date observed influence of catheter ablation techniques on autonomic regulation, a comprehensive method for evaluating the intraprocedural changes in parasympathetic activity following PVI is still lacking. Parasympathetic GPs, which are typically situated in close proximity to the ostia of pulmonary veins [29]—the primary target during PVI—can be frequently modified during AF ablation procedure. One of the two major GPs is located in close vicinity to the ostium of the RSPV and left atrium—the superior paraseptal GP (SPSGP) [30] which is considered to play the most significant role in the parasympathetic innervation of the sinoatrial node [31]. The application of RF energy during PVI, vital for achieving electrical isolation of the pulmonary veins, can impact SPSGP and adjacent GPs [23]. This unintentional vagal denervation (cardioneuromodulation), especially in the SPSGP region, may contribute significantly to the observed alterations in HR and autonomic markers such as ST-DC and ST-HRV. Prior research has described associations between DC and HRV in the context of PVI [32–34]. However, there are still limited data in the literature concerning intraprocedural recordings, such as ST-DC and ST-HRV. The use of ST-DC and ST-HRV offers possibility of non-invasive pre-procedural assessment of vagal tone, among others before and after PVI. While traditional 24-h recordings have long been employed to study autonomic modulation [32, 33], short-term recordings can offer distinct advantages, especially in dynamic measurements during PVI. The immediate availability of these recordings combined with the precise calculation of R-R intervals using electrophysiological systems allows for a detailed capture of immediate autonomic changes and analysis of vagal activity during critical stages of the ablation procedure. In the context of PVI, where frequently inadvertent cardioneuroablation/modulation of GP takes place, the temporal specificity of recordings appears particularly relevant. Such a cardioneuromodulative effect indicates a need for real-time assessment of autonomic responses to guide procedural decisions and post-PVI management effectively [30]. Traditional 24-h recordings, while valuable for assessing baseline autonomic function, may lack the precision needed to capture subtle changes induced by interventions like PVI, especially during the immediate post-PVI period. ST-DC and ST-HRV obtained from 1-min ECG recordings, which were investigated in the current study, can overcome some of the limitations listed above and offer a non-invasive, quick, and inexpensive tool for monitoring vagal tone [17]. Nevertheless, the optimal time of ECG monitoring for ST-DC and ST-HRV calculations still needs to be determined in future studies. Another consideration is the impact of antiarrhythmic drugs (AADs) and β-blockers, which are frequently used in patients with AF, on HRV and DC. Various studies have reported conflicting results regarding their influence on these parameters, and there is still a lack of consensus in this area. Some research has suggested that AADs and β-blockers can significantly affect HRV and DC, potentially altering the autonomic balance [35–38]. Conversely, other studies have found no significant impact of AADs and β-blockers on these autonomic markers [33, 39, 40]. Impact of AADs and β-blockers on ST-DC and ST-HRV has not been investigated so far.

This study demonstrated a trend that patients with enhanced parasympathetic activity, as indicated by a baseline ST-DC greater than 7.5 ms, are less likely to experience AF recurrence during the initial 3-month post-PVI observation period. This phenomenon can be attributed to the acute decrease in vagal tone immediately following PVI, as a result of inadvertent GP cardioneuromodulation. The reduced parasympathetic activity can create a less favorable environment for AF initiation and maintenance during this early post-procedural period [41], likely due to impaired likelihood for potentially proarrhythmic bradycardiac fluctuations [42]. The observed lack of sustained freedom from AF in patients with initially altered ST-DC beyond 12 months can be explained by reinnervation phenomenon, leading to a restoration of autonomic tone [43, 44]. Those individuals with initially enhanced parasympathetic activity may initially benefit from cardioneuromodulation effect; however, with time, reinnervation effect can lead to increased vulnerability to arrhythmia recurrence, potentially explaining the convergence of AF recurrence rates during extended follow-up period. This phenomenon may occur especially after standard thermal PVI, when complete cardioneuroablation of GP is not performed. Finally, with the advent of new non-thermal techniques such as pulsed field ablation (PFA), it would be interesting to see their impact on acute dynamic indicators of parasympathetic activity, including ST-DC. Initial studies indicate that PFA has only a short-lasting and limited effect on cardiac autonomic innervation [28, 45–47] with this impact being notably smaller and of shorter duration compared to cryoballoon ablation [48]. Moreover, the efficiency and safety of epicardial GPs ablation using PFA have shown promising results, indicating that this method may be useful and offer low risk of periprocedural complications [49]. However, despite these findings, the specific influence of PFA on dynamic markers remains unexplored. In addition to investigating the potential impact of PFA on parasympathetic activity, it can be necessary to determine its influence on parameters such as ST-DC and ST-HRV. Current data lacks investigations on how the PFA techniques affect these non-invasive autonomic markers.

### Limitations

This study has several limitations. First, the study group is small, and thus the results may be treated only as hypothesis-generating. Second, the recording time is shorter than performed in some previous studies [50–52], and there is an absence of continuous monitoring beyond the immediate post-PVI period which restricts the ability to capture the dynamic and potentially delayed changes in ST-DC and ST-HRV. Thirdly, PVI was performed with two different techniques which could have an impact on ST-DC and ST-HRV. Additionally, extracardiac vagal stimulation was not used to acutely assess the level of parasympathetic denervation. Moreover, AAD discontinuation protocol has not been standardized in our study; thus, the intake of these drugs could have some impact on ST-DC and ST-HRV measurements. Finally, the study’s 12-month follow-up may not capture longer-term autonomic remodelling dynamics.

## Conclusions

Measurement of ST-DC and ST-HRV before and after PVI is feasible. Patients with increased baseline ST-DC have reduced likelihood of AF recurrence during the short-term follow-up. Properly designed studies with larger cohorts are needed to validate the prognostic value of autonomic markers in patients undergoing PVI.

## Data Availability

The data presented in this study are available from the corresponding author upon request.

## Abbreviations

AAD: Antiarrhythmic drugs
AF: Atrial fibrillation
ECVS: Extracardiac vagal stimulation
GP: Ganglionated plexus
HF: High frequency
HRV: Heart rate variability
LA: Left atrium
LF: Low frequency
PBP: Point-by-point
PFA: Pulsed field ablation
PNN50: Proportion of success normal-to-normal RR intervals differing more than 50 ms divided by total number of normal-to-normal RR intervals
PVI: Pulmonary vein isolation
RMSSD: Root mean square of successive differences
SDNN: Standard deviation of normal-to-normal RR intervals
SPSGP: Superior paraseptal ganglionated plexus
ST-DC: Short-term deceleration capacity
ST-HRV: Short-term heart rate variability
VLF: Very low frequency

## References

[1] Camm AJ, et al. 2012 focused update of the ESC guidelines for the management of atrial fibrillation: an update of the 2010 ESC guidelines for the management of atrial fibrillation—developed with the special contribution of the European Heart Rhythm Association. Europace. 2012;14(10):1385–413. 10.1093/europace/eus305. 22923145 10.1093/europace/eus305

[2] Chen S, et al. Evolving role of catheter ablation for atrial fibrillation: early and effective rhythm control. J Clin Med. 2022;11(22):6871. 10.3390/jcm11226871. 36431348 10.3390/jcm11226871 PMC9696051

[3] Rebecchi M, et al. Atrial fibrillation and sympatho–vagal imbalance: from the choice of the antiarrhythmic treatment to patients with syncope and ganglionated plexi ablation. Eur Heart J Suppl. 2023;25(Suppl C):C1–6. 10.1093/eurheartjsupp/suad075. 37125283 10.1093/eurheartjsupp/suad075 PMC10132557

[4] de Vos CB, et al. Autonomic trigger patterns and anti-arrhythmic treatment of paroxysmal atrial fibrillation: data from the Euro Heart Survey. Eur Heart J. 2008;29(5):632–9. 10.1093/eurheartj/ehn025. 18270212 10.1093/eurheartj/ehn025

[5] Xi Y, Cheng J. Dysfunction of the autonomic nervous system in atrial fibrillation. J Thorac Dis. 2015;7(2):193–8. 10.3978/j.issn.2072-1439.2015.01.12. 25713736 10.3978/j.issn.2072-1439.2015.01.12 PMC4321068

[6] Rackley J, Nudy M, Gonzalez MD, Naccarelli G, Maheshwari A. Pulmonary vein isolation with adjunctive left atrial ganglionic plexus ablation for treatment of atrial fibrillation: a meta-analysis of randomized controlled trials. J Interv Card Electrophysiol. 2023;66(2):333–42. 10.1007/s10840-022-01212-1. 35419670 10.1007/s10840-022-01212-1

[7] Katritsis DG, et al. Autonomic denervation added to pulmonary vein isolation for paroxysmal atrial fibrillation: a randomized clinical trial. J Am Coll Cardiol. 2013;62(24):2318–25. 10.1016/j.jacc.2013.06.053. 23973694 10.1016/j.jacc.2013.06.053

[8] Pokushalov E, et al. Ganglionated plexus ablation vs linear ablation in patients undergoing pulmonary vein isolation for persistent/long-standing persistent atrial fibrillation: a randomized comparison. Heart Rhythm. 2013;10(9):1280–6. 10.1016/j.hrthm.2013.04.016. 23608592 10.1016/j.hrthm.2013.04.016

[9] Kampaktsis PN, Oikonomou EK, Choi DY, Cheung JW. Efficacy of ganglionated plexi ablation in addition to pulmonary vein isolation for paroxysmal versus persistent atrial fibrillation: a meta-analysis of randomized controlled clinical trials. J Interv Card Electrophysiol. 2017;50(3):253–60. 10.1007/s10840-017-0285-z. 28887742 10.1007/s10840-017-0285-z

[10] Lewis MJ. Heart rate variability analysis: a tool to assess cardiac autonomic function. Comput Inform Nurs. 2005;23(6):335–41. 10.1097/00024665-200511000-00011. 16292049 10.1097/00024665-200511000-00011

[11] Chen W, et al. Extracardiac Vagal stimulation-assisted cardioneuroablation: dynamically evaluating the impact of sequential ganglionated plexus ablation on vagal control of SAN and AVN in patients with sinoatrial node dysfunction. J Cardiovasc Dev Dis. 2022;9(6):188. 10.3390/jcdd9060188. 35735817 10.3390/jcdd9060188 PMC9225033

[12] Piotrowski R, Zuk A, Baran J, Sikorska A, Krynski T, Kulakowski P. Ultrasound-guided extracardiac vagal stimulation—new approach for visualization of the vagus nerve during cardioneuroablation. Heart Rhythm. 2022;19(8):1247–52. 10.1016/j.hrthm.2022.04.014. 35462051 10.1016/j.hrthm.2022.04.014

[13] Pachon-M EI, et al. Relation of fractionated atrial potentials with the vagal innervation evaluated by extracardiac vagal stimulation during cardioneuroablation. Circ: Arrhythmia Electrophysiol. 2020;13(4). 10.1161/CIRCEP.119.007900. 10.1161/CIRCEP.119.007900 32188285

[14] Orini M, et al. Long-term association of ultra-short heart rate variability with cardiovascular events. Sci Rep. 2023;13(1). 10.1038/s41598-023-45988-2. 10.1038/s41598-023-45988-2 PMC10624663 37923787

[15] Thong T, Li K, McNames J, Aboy M, Goldstein B. Accuracy of ultra-short heart rate variability measures. In: Proceedings of the 25th Annual International Conference of the IEEE Engineering in Medicine and Biology Society (IEEE Cat. No.03CH37439). 2003. pp. 2424–2427 Vol.3. 10.1109/IEMBS.2003.1280405.

[16] Esco MR, Flatt AA. Ultra-short-term heart rate variability indexes at rest and post-exercise in athletes: evaluating the agreement with accepted recommendations. J Sports Sci Med. 2014;13(3):535–41. 25177179 PMC4126289

[17] Baek HJ, Cho C-H, Cho J, Woo J-M. Reliability of ultra-short-term analysis as a surrogate of standard 5-min analysis of heart rate variability. Telemed J E Health. 2015;21(5):404–14. 10.1089/tmj.2014.0104. 25807067 10.1089/tmj.2014.0104

[18] Munoz ML, et al. Validity of (ultra-)short recordings for heart rate variability measurements. Plos one. 2015;10(9):e0138921. 10.1371/journal.pone.0138921. 26414314 10.1371/journal.pone.0138921 PMC4586373

[19] Wu L, Shi P, Yu H, Liu Y. An optimization study of the ultra-short period for HRV analysis at rest and post-exercise. J Electrocardiol. 2020;63:57–63. 10.1016/j.jelectrocard.2020.10.002. 33142181 10.1016/j.jelectrocard.2020.10.002

[20] Zheng L, et al. The diagnostic value of cardiac deceleration capacity in vasovagal syncope. Circ: Arrhythmia Electrophysiol. 2020;13(12):e008659. 10.1161/CIRCEP.120.008659. 10.1161/CIRCEP.120.008659 33197331

[21] Futyma P, Ciąpała K, Sander J, Głuszczyk R, Futyma M, Kułakowski P. Ultrasound-guided venous access facilitated by the Valsalva maneuver during invasive electrophysiological procedures. Kardiol Pol. 2020;78(3):235–9. 10.33963/KP.15188. 32049071 10.33963/KP.15188

[22] Stavrakis S, Nakagawa H, Po SS, Scherlag BJ, Lazzara R, Jackman WM. The role of the autonomic ganglia in atrial fibrillation. JACC: Clin Electrophysiol. 2015;1(1):1–13. 10.1016/j.jacep.2015.01.005. 26301262 10.1016/j.jacep.2015.01.005 PMC4540352

[23] Tang LYW, et al. Autonomic alterations after pulmonary vein isolation in the CIRCA-DOSE (cryoballoon vs irrigated radiofrequency catheter ablation) study. J Am Heart Assoc. 2021;10(5):e018610. 10.1161/JAHA.120.018610. 33634706 10.1161/JAHA.120.018610 PMC8174287

[24] Musikantow DR, et al. Pulsed field ablation to treat atrial fibrillation: autonomic nervous system effects. JACC Clin Electrophysiol. 2023;9(4):481–93. 10.1016/j.jacep.2022.10.028. 36752473 10.1016/j.jacep.2022.10.028

[25] Sikorska A, et al. Acceleration of sinus rhythm following ablation for atrial fibrillation: a simple parameter predicting ablation efficacy. Kardiol Pol. 2019;77(10):960–5. 10.33963/KP.14950. 31456591 10.33963/KP.14950

[26] Maciejewski C, et al. Is increased resting heart rate after radiofrequency pulmonary vein isolation a predictor of favorable long-term outcome of the procedure? J Clin Med. 2022;11(8):2159. 10.3390/jcm11082159. 35456252 10.3390/jcm11082159 PMC9025177

[27] Wagner L, et al. Cryoballoon pulmonary vein isolation-mediated rise of sinus rate in patients with paroxysmal atrial fibrillation. Clin Res Cardiol. 2021;110(1):124–35. 10.1007/s00392-020-01659-0. 32405738 10.1007/s00392-020-01659-0 PMC7806555

[28] Stojadinović P, et al. Autonomic changes are more durable after radiofrequency than pulsed electric field pulmonary vein ablation. JACC Clin Electrophysiol. 2022;8(7):895–904. 10.1016/j.jacep.2022.04.017. 35863816 10.1016/j.jacep.2022.04.017

[29] Avazzadeh S, et al. Ganglionated plexi ablation for the treatment of atrial fibrillation. J Clin Med. 2020;9(10):3081. 10.3390/jcm9103081. 32987820 10.3390/jcm9103081 PMC7598705

[30] Osório TG, et al. Standardized quantification of vagal denervation by extracardiac vagal stimulation during second generation cryoballoon ablation: a vein per vein analysis. J Atr Fibrillation. 2019;12(3):2223. 10.4022/jafib.2223. 32435337 10.4022/jafib.2223 PMC7237095

[31] Hu F, et al. Right anterior ganglionated plexus: the primary target of cardioneuroablation? Heart Rhythm. 2019;16(10):1545–51. 10.1016/j.hrthm.2019.07.018. 31330187 10.1016/j.hrthm.2019.07.018

[32] Bauer A, et al. Effects of circumferential or segmental pulmonary vein ablation for paroxysmal atrial fibrillation on cardiac autonomic function. Heart Rhythm. 2006;3(12):1428–35. 10.1016/j.hrthm.2006.08.025. 17161785 10.1016/j.hrthm.2006.08.025

[33] Chen Z, et al. Low heart deceleration capacity imply higher atrial fibrillation-free rate after ablation. Sci Rep. 2018;8:5537. 10.1038/s41598-018-23970-7. 29615802 10.1038/s41598-018-23970-7 PMC5883009

[34] Aksu T, et al. The impact of the clinical diagnosis on the vagal response and heart rate after ganglionated plexus ablation. J Interv Card Electrophysiol. 2022. 10.1007/s10840-022-01270-5. 35752732 10.1007/s10840-022-01270-5

[35] Niehoff J, Matzkies M, Nguemo F, Hescheler J, Reppel M. The effect of antiarrhythmic drugs on the beat rate variability of human embryonic and human induced pluripotent stem cell derived cardiomyocytes. Sci Rep. 2019;9(1):14106. 10.1038/s41598-019-50557-7. 31575920 10.1038/s41598-019-50557-7 PMC6773847

[36] Boonhoh W, Kijtawornrat A, Sawangkoon S. Comparative effects of amiodarone and dronedarone treatments on cardiac function in a rabbit model. Vet World. 2019;12(2):345–51. 10.14202/vetworld.2019.345-351. 31040580 10.14202/vetworld.2019.345-351 PMC6460874

[37] Lombardi F, et al. Beta-blocking effect of propafenone based on spectral analysis of heart rate variability. Am J Cardiol. 1992;70(11):1028–34. 10.1016/0002-9149(92)90355-3. 1357951 10.1016/0002-9149(92)90355-3

[38] Niemelä MJ, Airaksinen KE, Huikuri HV. Effect of beta-blockade on heart rate variability in patients with coronary artery disease. J Am Coll Cardiol. 1994;23(6):1370–7. 10.1016/0735-1097(94)90379-4. 8176095 10.1016/0735-1097(94)90379-4

[39] Hu W, et al. Deceleration and acceleration capacities of heart rate associated with heart failure with high discriminating performance. Sci Rep. 2016;6:23617. 10.1038/srep23617. 27005970 10.1038/srep23617 PMC4804298

[40] Sanderson JE, et al. Beta-blockade in heart failure: a comparison of carvedilol with metoprolol. J Am Coll Cardiol. 1999;34(5):1522–8. 10.1016/S0735-1097(99)00367-8. 10551702 10.1016/s0735-1097(99)00367-8

[41] Park H-W, Shen MJ, Lin S-F, Fishbein MC, Chen LS, Chen P-S. Neural mechanisms of atrial fibrillation. Curr Opin Cardiol. 2012;27(1):24–8. 10.1097/HCO.0b013e32834dc4e8. 22139702 10.1097/HCO.0b013e32834dc4e8 PMC3279730

[42] Gorenek B. Clinical importance of short-long-short sequences: analysing the mode of onset of ventricular tachycardias and atrial fibrillation. Int J Cardiol. 2009;137(2):177–80. 10.1016/j.ijcard.2008.05.032. 18674827 10.1016/j.ijcard.2008.05.032

[43] Sakamoto S, Schuessler RB, Lee AM, Aziz A, Lall SC, Damiano RJ. Vagal denervation and reinnervation after ablation of ganglionated plexi. J Thorac Cardiovasc Surg. 2010;139(2):444. 10.1016/j.jtcvs.2009.04.056. 19740492 10.1016/j.jtcvs.2009.04.056 PMC2813372

[44] Aksu T, Skeete JR, Huang HH. Ganglionic plexus ablation: a step-by-step guide for electrophysiologists and review of modalities for neuromodulation for the management of atrial fibrillation. Arrhythmia Electrophysiol Rev. 2023;12. 10.15420/aer.2022.37. 10.15420/aer.2022.37 PMC9945432 36845167

[45] Tohoku S, et al. Impact of pulsed-field ablation on intrinsic cardiac autonomic nervous system after pulmonary vein isolation. JACC Clin Electrophysiol. 2023;9(9):1864–75. 10.1016/j.jacep.2023.05.035. 37480870 10.1016/j.jacep.2023.05.035

[46] Guo F, et al. Effects of pulsed field ablation on autonomic nervous system in paroxysmal atrial fibrillation: a pilot study. Heart Rhythm. 2023;20(3):329–38. 10.1016/j.hrthm.2022.11.013. 36435350 10.1016/j.hrthm.2022.11.013

[47] Del Monte A, et al. Pulsed field ablation of the right superior pulmonary vein prevents vagal responses via anterior right ganglionated plexus modulation. Heart Rhythm. 2024;21(6):780–7. 10.1016/j.hrthm.2024.01.040. 38290688 10.1016/j.hrthm.2024.01.040

[48] Del Monte A, et al. Quantitative assessment of transient autonomic modulation after single-shot pulmonary vein isolation with pulsed-field ablation. J Cardiovasc Electrophysiol. 2023;34(11):2393–7. 10.1111/jce.16089. 37792572 10.1111/jce.16089

[49] Musikantow DR, et al. Targeted ablation of epicardial ganglionated plexi during cardiac surgery with pulsed field electroporation (NEURAL AF). J Interv Card Electrophysiol. 2023. 10.1007/s10840-023-01615-8. 37561246 10.1007/s10840-023-01615-8 PMC12043773

[50] Rizas KD, et al. Bedside autonomic risk stratification after myocardial infarction by means of short-term deceleration capacity of heart rate. EP Europace. 2018;20(FI1):f129–36. 10.1093/europace/eux167. 29106527 10.1093/europace/eux167

[51] Sacha J, Sobon J, Sacha K, Muller A, Schmidt G. Short-term deceleration capacity reveals higher reproducibility than spectral heart rate variability indices during self-monitoring at home. Int J Cardiol. 2011;152(2):271–2. 10.1016/j.ijcard.2011.08.008. 21880384 10.1016/j.ijcard.2011.08.008

[52] Okwose NC, et al. Validity and reliability of short-term heart-rate variability from disposable electrocardiography leads. Health Science Reports. 2023;6(1):e984. 10.1002/hsr2.984. 36514326 10.1002/hsr2.984 PMC9731360

